# A method for estimation of accuracy of dose delivery with dynamic slit windows in medical linear accelerators

**DOI:** 10.4103/0971-6203.42768

**Published:** 2008

**Authors:** R. Ravichandran, J. P. Binukumar, S. S. Sivakumar, K. Krishnamurthy, C. A. Davis

**Affiliations:** Medical Physics Unit, Department of Radiotherapy, National Oncology Center, Royal Hospital, Muscat, Sultanate of Oman

**Keywords:** Accuracy dose delivery, quality assurance methods, sliding window intensity-modulated radiotherapy

## Abstract

Intensity-modulated radiotherapy (IMRT) clinical dose delivery is based on computer-controlled multileaf movements at different velocities. To test the accuracy of modulation of the beam periodically, quality assurance (QA) methods are necessary. Using a cylindrical phantom, dose delivery was checked at a constant geometry for sweeping fields. Repeated measurements with an in-house designed methodology over a period of 1 year indicate that the method is very sensitive to check the proper functioning of such dose delivery in medical linacs. A cylindrical perspex phantom with facility to accurately position a 0.6-cc (FC 65) ion chamber at constant depth at isocenter, (SA 24 constancy check tool phantom for MU check, Scanditronix Wellhofer) was used. Dosimeter readings were integrated for 4-mm, 10-mm, 20-mm sweeping fields and for 3 angular positions of the gantry periodically. Consistency of standard sweeping field output (10-mm slit width) and the ratios of outputs against other slit widths over a long period were reported. A 10-mm sweeping field output was found reproducible within an accuracy of 0.03% (n = 25) over 1 year. Four-millimeter, 20-mm outputs expressed as ratio with respect to 10-mm sweep output remained within a mean deviation of 0.2% and 0.03% respectively. Outputs at 3 gantry angles remained within 0.5%, showing that the effect of dynamic movements of multileaf collimator (MLC) on the output is minimal for angular positions of gantry. This method of QA is very simple and is recommended in addition to individual patient QA measurements, which reflect the accuracy of dose planning system. In addition to standard output and energy checks of linacs, the above measurements can be complemented so as to check proper functioning of multileaf collimator for dynamic field dose delivery.

## Introduction

High accuracy in clinical dose delivery is demanded for intensity-modulated radiotherapy (IMRT). Sliding window technique with multileaf collimator (MLC) leaves is one of the most commonly used techniques of dose delivery with IMRT in the recent past. During motion of the individual leaves, the velocity of movements decides the total dose delivered in a single sequence. With different gantry orientations, there may be gravity effects on the movements of slit diaphragms. Therefore, there is need to have periodical measurements and quality assurance (QA) methods 1) to confirm dose delivery of dynamic field output at isocenter of linear accelerators for different gantry angles; and 2) to confirm reproducibility in outputs for small- and medium-sized sweeping fields, which decide the accuracy in dose delivered at the desired depth in a patient. A method designed to achieve the above-mentioned objectives is described in this technical note.

## Materials and Methods

**Figure 1:**
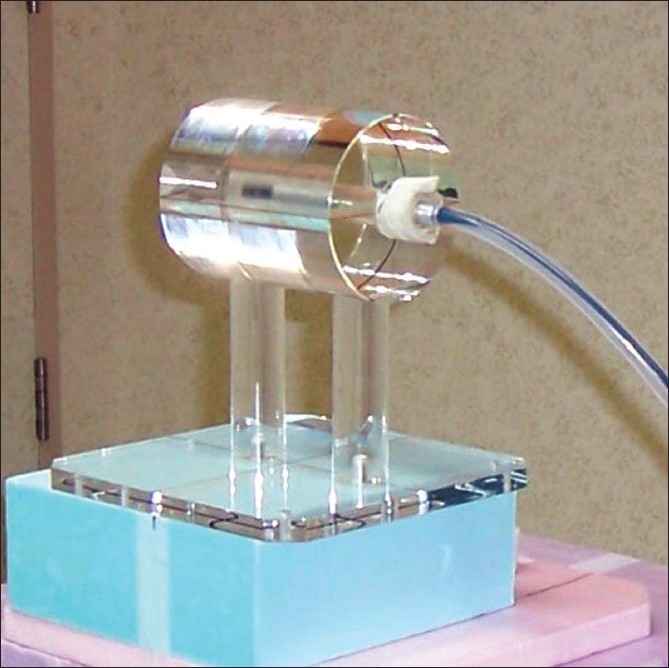
The cylindrical test tool to provide a constant depth of 5 cm for an FC65 ion chamber. Three angulations of the gantry can be checked with this tool routinely. Under-table gantry position cannot be measured as the mounting block comes in the path of the beam

A constant-depth cylindrical perspex phantom, with facility to accurately position a 0.6-cc (FC 65) ion chamber (SA 24 constancy check tool for MU check, Scanditronix Wellhofer) was used in this study [Figure 1]. The cylindrical phantom has a diameter of 10 cm, providing a depth of 5 cm at the isocenter. Measurements were done on a 120-leaf, 100-cm Source Axis Distance (SAD), Varian Clinac-2300 CD linear accelerator, Varian Clinac-2300 CD linear accelerator.

### Checking of output consistency with sliding window at different angles

**Figure 2:**
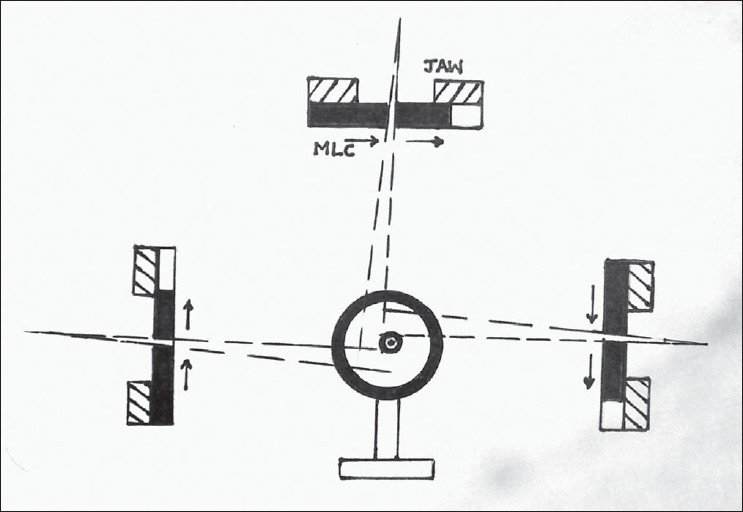
Geometry of the ion chamber to integrate the dose for a moving slit field of variable widths. The directions of the leaf movements differ for each orientation of the gantry. Above the level of MLC leaves, jaw size has pre-selectable openings for each sliding window field

Output consistency for 200 MU for a 10-mm sweeping field for 6-MV photon over a field size of 10×10 cm obtainable with MLC was studied. This corresponded to a standard jaw setting of X = 11.6 cm; Y = 20.4 cm. Nine readings were obtained with Dose1 electrometer (Scanditronix Wellhofer), 3 readings each for 0°, 90°, 270° gantry angles. Standard deviations were expressed for the 9 readings, and the mean reading was corrected for daily variations of room temperature and atmospheric pressure. These tests were repeated at alternate weeks. Geometry of measurements is shown in Figure 2.

### Small- and large-size sweep field outputs

At gantry angle 0°, sweeping field outputs for sliding windows 4 mm and 20 mm were measured for 200 MU. Mean values of integrated dosimeter readings for these sweep fields were expressed as ratios of outputs 4 mm/10 mm and 10 mm/20 mm, to represent proper radiation dose delivery with these fields.

### Reference values for the department

The values obtained as mean of 10 measurements over a period of 3 months (February-May 2007) were taken as standard for future reference. The standard deviations obtained for these measurements are expected to represent the reproducibility of these data relating to the proper functioning of the moving parts in the linac.

## Result

Corrected readings for mean of outputs (200 MU) for 10-mm sweeping field, mean of their standard deviations, mean ratios of 4 mm/10 mm and 10 mm/20 mm outputs are shown in Table 1. The observed trend of these measured values of test parameters for the linac for a period of 1 year (n = 25) is shown in Table 2.

**Table 1:** Standard values obtained during the first 3 months

*10mm slit output (200MU)*	*Standard deviation for 3 gantry angles 0°, 90°, 270°*	*Ratio of outputs for 4mm, 10mm*	*Ratio of outputs for 10mm, 20mm*
246.2+1.0 (0.4%)	0.34 % +0.1	0.602 + 0.0012	0.630+ 0.0014

**Table 2:** Stability of test parameters of dynamic field dose delivery

*Mean deviation in 10 mm sweep output (%)*	*Stability in standard deviations of 3 gantry angle outputs*	*Measured output ratios 4mm/10mm Mean deviation %*	*Measured output ratios 10mm/20mm Mean deviation%*
−0.025+0.71	Within 0.5% SD	0.25+0.71	0.03+0.21

## Discussion

The method presented in this technical note may be useful for routine quality assurance of the dynamic field output delivery of the medical linear accelerators used for delivery of IMRT treatments. There are no specific protocols found in literature for such routine checks of dynamic treatment delivery. In our department, we have recently introduced IMRT treatments for the patients[1] and there was an immediate need for implementation of a simple method for routine QA for linac, in addition to weekly output measurements for standard fields. It can be seen [Table 2] that the sweeping field output was highly consistent within a mean deviation of 0.03%. The 3 gantry angles represent movements of MLC leaves in different orientations. However, the standard deviations of 9 readings (3 for each angle) were within 0.5% over a long duration of 1 year (n = 25).

Mean deviation of 4-mm sweep output against 10-mm sweep output was 0.25%, which is slightly higher than the 10 mm/20 mm sweep outputs ratio of 0.03%. This is as expected, because maintaining very small (4 mm) opening by continuous motor movements mechanically is much difficult to achieve than 20-mm sweep widths. Moreover, 4-mm slit openings are not precisely planned by the treatment planning system also. The present-generation linacs are digitally controlled, and the results of the present work reveal the ideal functioning of the linac for dynamic dose delivery.

## Conclusion

QA methodology suggested in the present work could be carried out periodically in the department as a complementary method to test stationary fields. It is believed that any changes in the linac dose delivery during dynamic sliding of window could be easily detected by this method. This method, however, checks only the composite dose at the isocenter from the dynamic slit window fields, but not the accuracy of individual field dose delivery. The method outlined in this work, therefore, does not replace the IMRT QA requirement for individual dose mapping for each patient plan.
